# Beliefs About COVID-19 in Canada, the United Kingdom, and the United States: A Novel Test of Political Polarization and Motivated Reasoning

**DOI:** 10.1177/01461672211023652

**Published:** 2021-06-28

**Authors:** Gordon Pennycook, Jonathon McPhetres, Bence Bago, David G. Rand

**Affiliations:** 1 University of Regina, Saskatchewan, Canada; 2 Durham University, Durham, UK; 3 University of Toulouse Capitole, France; 4 Massachusetts Institute of Technology, Cambridge, USA

**Keywords:** COVID-19, motivated reasoning, political polarization, cognitive reflection, attitudes

## Abstract

What are the psychological consequences of the increasingly politicized nature of the COVID-19 pandemic in the United States relative to similar Western countries? In a two-wave study completed early (March) and later (December) in the pandemic, we found that polarization was greater in the United States (*N* = 1,339) than in Canada (*N* = 644) and the United Kingdom. (*N* = 1,283). Political conservatism in the United States was strongly associated with engaging in weaker mitigation behaviors, lower COVID-19 risk perceptions, greater misperceptions, and stronger vaccination hesitancy. Although there was some evidence that cognitive sophistication was associated with increased polarization in the United States in December (but not March), cognitive sophistication was nonetheless consistently negatively correlated with misperceptions and vaccination hesitancy across time, countries, and party lines. Furthermore, COVID-19 skepticism in the United States was strongly correlated with distrust in liberal-leaning mainstream news outlets and trust in conservative-leaning news outlets, suggesting that polarization may be driven by differences in information environments.

## Introduction

There have been numerous news reports indicating that many people have not been taking the coronavirus disease 2019 (COVID-19) disease seriously. Furthermore, there is evidence that misinformation about COVID-19 has proliferated (Loomba et al., 2021), suggesting that belief in harmful misperceptions may be contributing to this problem (Enders et al., 2020). Given that having even a vaguely accurate understanding of the risk posed by COVID-19 is presumably a central first step toward mobilizing the behavior changes necessary to save lives, it is, therefore, important to understand why people differ in their opinions about COVID-19. This also provides an opportunity to test psychological theories about political polarization and science beliefs.

## Political Partisanship

One of the most salient apparent sources of disagreement surrounding COVID-19 is political partisanship. In the United States, President Donald Trump has vacillated on the level of threat that the nation is facing. Although Trump declared a state of emergency on March 13, 2020, he has been notably skeptical about the risk posed by the virus. For example, he said that the country is “in very good shape” on February 14, likened it to the common flu on March 9, and, on March 24 when it was clear that the virus has spread throughout the country, he nonetheless proposed that the United States be “reopened” by Easter 2020 (a proposal he later backtracked on). Similar messaging has come from other elite Republicans and conservative media outlets such as Fox News (Simonov et al., 2020).

Interestingly, COVID-19 represents a sort of natural experiment: An identical crisis (in form) is being faced by other culturally similar countries, but without the seemingly extreme politicization that has occurred in the United States. For example, although there has been disagreement about the effectiveness and quality of Prime Minister Boris Johnson’s response to the pandemic in the United Kingdom, Johnson has nonetheless primarily deferred to experts. This stands in stark contrast to Trump, who has frequently contradicted even his own experts.

A more ambiguous comparison to the polarization in the United States comes from Canada. Prime Minister Justin Trudeau has not faced the same level of criticism as Trump. Furthermore, an early study of Twitter behavior from Canadian Member’s of Parliament found no evidence that members from any party were downplaying the pandemic (Merkley et al., 2020). However, so-called “culture wars” in the United States tend to spill over into Canadian politics and there is evidence that political ideology (although not political party membership) correlates with perceptions of COVID-19 severity in Canada (with conservatives viewing it as less severe; Merkley et al., 2020).

These apparent cross-country differences allow us to test whether political ideology per se (i.e., across countries) is associated with beliefs about COVID-19 (and, notably, rejection or skepticism of it) or whether this is relatively unique to the polarized political environment of the United States. Furthermore, the change in beliefs about COVID-19 over time should reveal increased polarization in the United States, in particular.

## Cognitive Sophistication

In addition to being a matter of public health and policy, COVID-19 is a scientific issue. Relevant experts and organizations such as the World Health Organization and American Centers for Disease Control and Prevention have warned extensively about the substantial risks to public health posed by COVID-19. Furthermore, many of the misperceptions about COVID-19 are similar to common misconceptions about science (and, in particular, medical science); for instance, that simple remedies (e.g., Vitamin C) are sufficient to cure diseases. Interestingly, the politicization of COVID-19 (at least in the United States) also brings it in parallel with other scientific topics. Thus, we also draw on research that focuses on science-related beliefs to better understand misperceptions about COVID-19.

Given the salience of politically divisive topics like climate change, it is perhaps unsurprising that research on why people believe what they believe about science has focused on the role of political ideology (e.g., Kahan et al., 2012; Mccright & Dunlap, 2011; Rutjens et al., 2018). There are a variety of theories that make somewhat different claims in this space, but a common feature is that anti-science beliefs are largely cultural. For example, historical evidence shows that conservative think-tanks actively politicized global warming (undermining scientists and relevant experts) (Brulle, 2013; Dunlap & Jacques, 2013; Jacques et al., 2008); this polluted information environment is then transmitted to the general public and interacts with the ideology of individuals such that political conservatives are motivated to accept anti-science attitudes (e.g., to protect their political identities; (Kahan et al., 2012, 2017).

Again, here, a comparison across countries where the cultural transmission of anti-science messaging seems to be differing (with greater skepticism about COVID-19 in the United States) allows for a novel test of this long-standing perspective. This is important because there has recently been some criticism of the focus on ideology as a causal factor in the context of science beliefs because it is likely that beliefs about science also proceed through what might be considered more “typical” information processing routes. For example, early theories about public understanding of science focused on the role of one’s basic science knowledge—referred to as knowledge deficit models. This perspective argues that people hold anti-science beliefs largely because they do not possess enough basic scientific knowledge to properly understand novel science-related claims (Allum et al., 2008; Sturgis & Allum, 2004). The claim that follows is that providing people with basic scientific knowledge will then lead to an increase in acceptance of science. In support of this, some recent research suggests that teaching people about the basic science behind genetically modified foods (McPhetres et al., 2019b) and climate change (Ranney & Clark, 2016) can actually lead to positive belief changes. Given that the current threat is a virus, it seems that fundamental biological and scientific knowledge might be especially relevant in assessing and responding to the threat.

Although the knowledge-deficit model has been criticized (Simis et al., 2016), research in other areas has converged on a similar conclusion: Not all reasoning is politically motivated and being more cognitively sophisticated (including, but not limited to, basic scientific knowledge) facilitates the adoption of accurate and pro-scientific beliefs. For example, people who are more reflective and analytic (as opposed to relying more on their intuitions) are more likely to endorse evolution (Gervais, 2015), and vaccination (Sarathchandra et al., 2018) and are less likely to believe in superstitions (Pennycook et al., 2012), conspiracies (Swami et al., 2014), and fake news (Pennycook & Rand, 2019b). Relatedly, people who are more receptive to pseudo-profound bullshit (i.e., they rate random sentences filled with buzzwords as profound) are more likely to believe in the efficacy of non-evidence-based alternative medicines and general conspiracy theories (Pennycook, Cheyne, et al., 2015) as well as fake news (Pennycook & Rand, 2020). Finally, being able to understand probabilities and numbers (i.e., numeracy; Peters et al., 2006) has been shown to be important for a variety of decisions, but most notably in medical contexts (Lipkus & Peters, 2009). Factors such as basic science knowledge, cognitive reflection, numeracy, and bullshit skepticism represent a set of related but unique cognitive competencies that apparently facilitate the adoption of pro-scientific beliefs. For simplicity, we will collectively refer to them as “cognitive sophistication” here.

Given the conflicting perspectives offered by this past research, it is unclear how cognitive processing will function in the context of COVID-19. In addition to helping to guide COVID-19 interventions, this may also shed light on the basic theoretical question of what shapes people’s beliefs about science, particularly as it relates to the relative roles of ideology versus cognitive sophistication (McPhetres et al., 2019a).

## Identity-Protective Cognition

Thus far, we have discussed the roles of cognitive sophistication and political polarization. However, there is another prominent theory that we have yet to address: identity-protective cognition (Drummond & Fischhoff, 2017; Kahan et al., 2011, 2012, 2017; Landrum et al., 2017). This account proposes that ideology and cognitive sophistication actually *interact* to predict attitudes about science and misinformation, such that individuals who have the strongest reasoning capacities are the *most* politically polarized (this is sometimes referred to as “motivated System 2 reasoning”). For example, whereas Democrats who are the most reflective and numerate believe that the risks associated with climate change are greater than those who are more intuitive and innumerate, the opposite pattern is evident among Republicans (Kahan et al., 2012). That is, the more cognitively sophisticated Republicans are actually *less* likely to hold beliefs that are consistent with the scientific consensus on climate change.

This research, however, faces the same criticism as research on the broad role of ideology on science beliefs: It tends to focus on a small number of issues and it is, therefore, unclear how generalizable it is to other scientific issues. Indeed, recent evidence indicates that there is very little evidence that cognitive sophistication is associated with *increased* anti-science attitudes, even in cases where such attitudes are politically congenial (McPhetres et al., 2019a; Pennycook et al., 2020). This research shows that, even if cognitive sophistication *interacts* with ideology when predicting some (but not all or even most) anti-science attitudes, the general pattern is that people who are better at reasoning are usually less likely to hold beliefs counter to scientific consensus. Furthermore, recent work in the context of political misinformation (“fake news”) shows that cognitive sophistication is associated with an increased capacity to discern between true and false content *regardless* of whether it is consistent or inconsistent with one’s political identity (Bago et al., 2020a; Pennycook & Rand, 2019b). This is consistent with the work cited above that shows the benefits of cognitive sophistication (and analytic thinking specifically) for accurate belief formation (Pennycook, Fugelsang, & Koehler, 2015).

To summarize, the COVID-19 crisis offers a unique possible test of two competing accounts: Does cognitive sophistication lead to more accurate beliefs overall or is it primarily used to support ideologically motivated reasoning and, therefore, increased political polarization?

## Study 1

For Study 1, we investigated these issues using three parallel preregistered surveys using quota-sampling of residents from the United States (*N* = 689) and the United Kingdom (*N* = 642), in addition to a convenience sample of residents from Canada (*N* = 644)—all via the polling firm *Prolific* (see Supplemental Materials, Table S1, for full demographic breakdown). The survey is, therefore, not nationally representative; however, the samples all came from the same source, which allows for a direct comparison. The survey was completed on March 24, 2020. Our data, materials, and preregistration are available on the Open Science Framework: OSF. All non-preregistered analyses are labeled as post hoc. We report all manipulations, measures, and exclusions in these studies.

## Method

### Participants

The U.K. and U.S. samples were recruited with quota-matching to approximate the national populations (via census data) across age, sex, and ethnicity. Prolific does not offer quota-matching for Canadian samples. In total, 753 (Canada), 765 (United Kingdom), and 759 (United States) entered the survey. However, some participants did not complete the survey (*N*’s = 5, 11, 17) and some did not indicate residing in the target country (*N*’s = 2, 1, 1). We also included three attention check questions (see OSF for full materials; Berinsky et al., 2014). Following our preregistration, we removed participants who failed two or more of these (*N*’s = 104, 111, 52). This left us with final sample sizes of 644 (Canada), 642 (United Kingdom), and 689 (United States).

### Materials and Procedure

Full materials and a copy of the Qualtrics survey file can be found on the OSF and descriptive statistics are available in the Supplemental Materials, Table S2. Measures are listed in order of presentation, unless otherwise stated. At the beginning of the study, participants were told that we had a number of questions about COVID-19, “the novel coronavirus that has recently been declared a global pandemic by the World Health Organization.” We then informed the participants that we will refer to COVID-19 as “coronavirus” throughout the survey for simplicity.

#### COVID-19 behavior change intentions

Participants were asked to rate the extent to which they intended to change their behavior in light of the coronavirus outbreak using a sliding scale from 0 (strongly disagree) to 100 (strongly agree) (Jordan et al., 2020). We first asked seven questions about stopping the spread through cleanliness (we did not mention the term cleanliness) (e.g., “wash my hands more often,” “stop hugging other people,” “try my hardest to avoid touching my face), followed by five questions related to sickness (e.g., “stay home if I am feeling even a little bit sick,” “cover my mouth when I cough and sneeze”). We then asked 10 questions about social distancing and, specifically, things that they were intending to avoid (e.g., “going to restaurants,” “going to the grocery store”). This was done on a scale from 0 (I will make no effort to avoid this activity) to 100 (I will completely avoid this activity). We also asked people to indicate which of these 10 activities they would engage in even if there was no coronavirus outbreak (e.g., some people may not go to restaurants regardless of the virus). We did not preregister any re-analysis of the data using this extra question, however. Indeed, the full behavior change intention measure was quite reliable across all three countries: Cronbach’s alpha = .86 (Canada), .85 (United Kingdom), and .88 (United States). At the very beginning of the study, participants were asked to select “67” on a sliding scale to ensure that their browser/device allowed them to use sliders. We exclude data for the intentions measure for two participants from the United Kingdom and two from the United States because they failed this check question (those who answered 66 or 68 were also retained, deviating from our preregistration).

#### COVID-19 risk perceptions

We asked eight questions related to risk perceptions (e.g., “The coronavirus poses a major threat to the public”). The scale had acceptable reliability in all three countries: Cronbach’s alpha = .73 (Canada), .77 (United Kingdom), and .83 (United States). We also asked three “personal” risk questions as an exploratory measure (e.g., “Because of my age and/or pre-existing conditions, I am likely to have serious symptoms if I were to contract the coronavirus”)—however, we did not preregister any analyses for this measure. Participants responded on a scale from 1 (strongly disagree) to 7 (strongly agree), with 4 (neither agree nor disagree) as the scale midpoint. We also included two exploratory questions about the trade-off between being coronavirus restrictions and the economy in the **Methodology File**. The risk perception questions were randomized with the misperception questions.

#### COVID-19 misperceptions

For misperceptions, we created a large list (k = 21) of falsehoods that have been spread about COVID-19 based on various news reports and fact-checking efforts. The misperceptions that we discovered fit broadly into four possible categories (see Table S3 in the Supplemental Materials), but we will focus on the overall misperception measure for simplicity (it was reliable in all three countries: Cronbach’s α = .80 [Canada], .79 [United Kingdom], and .84 [United States]). Although each falsehood was not believed by a particularly large proportion of individuals (with a few exceptions), most participants held at least one misperception (61% in Canada, 69% in the United Kingdom, and 66% in the United States). A full breakdown of the individual items can be found in Supplemental Materials, Table S3.

#### National leadership

Finally, we asked people 10 questions about how happy they are with the leadership of their respective countries. For brevity, we do not report these results here but the items can be found on OSF.

#### Cognitive sophistication

The four cognitive sophistication measures were presented in a random order for each participant. After each of the three tests (i.e., excluding the bullshit receptivity measure), participants were asked to estimate their accuracy.

We assessed science knowledge using 17 true/false question test (McPhetres et al., 2019a) with questions like “Electrons are smaller than atoms” and “Antibiotics kill viruses as well as bacteria” (this was the only item with direct relevance for COVID-19). The science knowledge test was sufficiently reliable in each country: Cronbach’s alpha =.74 (Canada), .74 (United Kingdom), and .77 (United States).

To assess the disposition to engage in analytic and reflective thinking, we used a 6-item Cognitive Reflection Test (CRT) (Frederick, 2005). The CRT consists of questions that trigger an automatic intuitive answer that is incorrect (e.g., “If you are running a race and pass the person in second place, what place are you in?”—the intuitive answer is the first place but the correct answer is the second place) and therefore requires reflection to override. Our measure consisted of a re-worded version of the original three items and three items from a non-numeric CRT (we excluded the “hole” item; Thomson & Oppenheimer, 2016). The CRT was sufficiently reliable in each country: Cronbach’s alpha =.70 (Canada), .70 (United Kingdom), and .72 (United States).

Numeracy was assessed using three items from the Berlin Numeracy Test (Cokely et al., 2012) and three items from the Lipkus numeracy scale (Lipkus et al., 2016). The questions all pertained to understanding probabilities that varied in difficulty (e.g., “Imagine we are throwing a five-sided die 50 times. On average, out of these 50 throws how many times would this five-sided die show an odd number [1, 3, or 5]?”). The numeracy test was sufficiently reliable in each country (albeit marginally so in Canada): Cronbach’s alpha =.65 (Canada), .71 (United Kingdom), and .73 (United States).

Finally, we included a measure of one’s general receptivity to bullshit (Pennycook, Cheyne, et al., 2015). This consisted of five sentences that were randomly using a corpus of buzzwords (e.g., “The invisible is beyond new timelessness”)—that is, pseudo-profound bullshit. Participants rated the profundity of the sentences on a 5-point scale from 1 (not at all profound) to 5 (very profound). This scale was then reverse-scored to put it in-line with the other cognitive sophistication measures.

For simplicity (and following our preregistration), we report the overall results with the omnibus measure of cognitive sophistication. For this, we *z*-scored each measure and took the mean of the four measures. The reliability of the four measure scale was similar to the reliabilities for the individual subscales in each country (using the 4 means as variables in the analysis): Cronbach’s alpha = .66 (Canada), .71 (United Kingdom), and .69 (United States). Alternatively, creating a composite measure of cognitive sophistication by simply taking each individual item from the subscales produces higher reliability (Cronbach’s α=.84 [Canada], .85 [United Kingdom], and .86 [United States]) but this does not equal weight the individual subscales. Thus, we report results for the omnibus measure that weighs each subscale equally.

#### Trust

As additional exploratory measures (their analyses were not included in the preregistration), we asked the participants to indicate their degree of trust in various information sources (e.g., social networking sites, scientists) using a 5-point scale from “none at all” to “a great deal.” We then asked them to indicate how much they trust various news sources specific to their own country. We created two composite variables based on the correlation between political conservatism (described below) and trust in the various news outlets. Those outlets that conservatives trusted more were coded as “conservative media” (United States: Fox News, Breitbart; United Kingdom: Daily Mail, The Times, The Daily Telegraph, and The Sun; Canada: Rebel Media) and those outlets that liberals trusted more were coded as “liberal media” (United States: CNN, MSNBC, New York Times, Washington Post, NPR, CBS News, ABC News, NBC News; United Kingdom: The Guardian, Channel 4; Canada: Global News, CBC, Toronto Star, Globe and Mail).

#### Political ideology

Participants in all three countries were then asked two questions about political ideology that were then combined to create our conservatism measure: “On social issues, I am: (1) Strongly Liberal, (2) Somewhat Liberal, (3) Moderate, (4) Somewhat Conservative, and (5) Strongly Conservative” and “On economic issues, I am: (1) Strongly Liberal, (2) Somewhat Liberal, (3) Moderate, (4) Somewhat Conservative, and (5) Strongly Conservative.”

In addition to the ideology measure, we also asked several country-specific questions. Participants in each country were asked about which federal party they align with (options changing depending on their country). They were also asked three questions about the national leadership in their respective country: (a) a feeling thermometer (0 = *extremely unfavorable feeling*; 100 = *extremely favorable feeling*), (b) strength of support or opposition (1 = *strongly oppose*; 7 = *strongly support*), and (c) likelihood of voting for the leader in the future (1 = *extremely unlikely*, 7 = *extremely likely*).

In the U.S. sample, we also included a continuous political partisanship measure where participants indicated their stance given the following options: Strongly Democratic, Democratic, Lean Democratic, Lean Republican, Republican, Strongly Republican. Notably, this was very highly correlated with political ideology, *r* = .82. Nonetheless, as post hoc robustness tests, we will report parallel analyses for partisanship where appropriate.

#### Demographics

We asked a variety of demographic questions at the end of the survey: age, education, income, gender, and general health.

## Results

### Political Ideology

To investigate the differential impact of political polarization, we correlated the same measure of political ideology (mean of social and economic liberal-conservatism) with our COVID-19 attitude and behavior measures across the three countries. As is evident from Table 1, political conservatism was associated with misperceptions in all three countries. However, this correlation was stronger in the United States than in the United Kingdom (correlations in Canada and the United States did not significantly differ, although the correlation was nominally larger in the United States; Table 2). The same pattern held for perceptions of COVID-19 risk and self-reported behavior change intentions: Political conservatives in the United States and Canada appear to have been taking COVID-19 less seriously than liberals, but the same pattern was not evident in the United Kingdom. The correlation between ideology and behavior change intentions was smaller but nonetheless divergent between the United States and the United Kingdom. Ideology was also a stronger predictor of COVID-19 risk perceptions in the United States than Canada once demographics (age, gender, income, education, and overall health) were controlled for (see Table S4 in Supplemental Materials—all other findings were identical with demographics included).

**Table 1.** Zero-Order Correlations Between Primary Measures in Canada (*N* = 644), United Kingdom (*N* = 642), and United States (*N* = 689).

Country	Variable	1	2	3	4	5	6
Canada	1. Misperceptions	—					
	2. COVID risk	−.34***	—				
	3. Change intentions	−.26***	.50***	—			
	4. Conservatism	.27***	−.26***	−.17***	—		
	5. Conservative media trust	.22***	−.14***	−.08	.17***	—	
	6. Liberal media trust	−.18***	.13***	.17***	−.21***	.09*	—
	7. Cognitive sophistication	−.34***	.002	−.03	−.11**	−.08*	.02
United Kingdom	1. Misperceptions	—					
	2. COVID risk	−.29***	—				
	3. Change intentions	−.10*	.44***	—			
	4. Conservatism	.14**	−.02	.07	—		
	5. Conservative media trust	.05	.06	.12**	.24***	—	
	6. Liberal media trust	−.20***	.23***	.15***	−.20***	.48***	—
	7. Cognitive sophistication	−.40***	−.11**	−.10*	−.19***	−.10***	.09*
United States	1. Misperceptions	—					
	2. COVID risk	−.40***	—				
	3. Change intentions	−.20***	.52***	—			
	4. Conservatism	.31***	−.36***	−.15***	—		
	5. Conservative media trust	.39***	−.26***	−.06	.55***	—	
	6. Liberal media trust	−.21***	.35***	.22***	−.45***	−.16***	—
	7. Cognitive sophistication	−.46***	.04	−.05	−.17***	−.25***	.03

*** *p* < .001. **p < .01. *p < .05. COVID = coronavirus disease.

**Table 2.** Multiple Regression Analyses (*B* and 95% Confident Intervals) Comparing the Correlation Between Key Dependent Variables and Ideology Across Countries (With the United States as a Baseline).

Country	Misperceptions	COVID risk	Change intentions
Conservatism	0.31***	−0.36***	−0.15***
	[0.24, 0.38]	[−0.43, −0.28]	[−0.23, −0.08]
Conservatism: United Kingdom	−0.17**	0.34***	0.22***
	[−0.28, −0.07]	[0.23, 0.44]	[0.11, 0.33]
Conservatism: Canada	−0.05	0.10	−0.02
	[−0.15, 0.06]	[−0.00, 0.21]	[−0.13, 0.09]
*N*	1,975	1,975	1,971
*R* ^2^	.06	.07	.02

*Note.* Conservatism was standardized (*z*-scored) within country prior to analysis. Canada (*N* = 644), United Kingdom (*N* = 642), and United States (*N* = 689). COVID = coronavirus disease.

Notably, a post hoc analysis found that trust in conservative news outlets in the United States and Canada (but not the United Kingdom) correlated significantly with higher misperceptions and weaker COVID risk perceptions (see Table 1). Trust in liberal news outlets in all three countries was associated with fewer misperceptions and higher COVID risk perceptions, suggesting that a potential source of polarization is specifically contrasting narratives in right-wing versus mainstream (“liberal-leaning”) media coverage in the United States and Canada. In fact, when liberal and conservative media trust are entered into a multiple regression analysis with conservatism in the U.S. sample, the former are significant predictors of misperceptions (liberal media trust: β = −.13, *p* = .001; conservative media trust: β = .34, *p* < .001) and political conservatism is no longer predictive (β = .07, *p* = .143).^1^ It should be noted, that the outlets in the “liberal” category, such as the New York Times and Washington Post in the United States and BBC and the Guardian in the United Kingdom, tend to be higher quality (Pennycook & Rand, 2019a). Thus, although causality cannot be determined with our cross-sectional design, differential exposure to low-versus-high quality content relating to COVID-19 may be a source of political polarization.

### Cognitive Sophistication

As is evident from Table 1, overall cognitive was a strong negative predictor of COVID-19 misperceptions (*r*’s = −.34, −.40, −.46 in Canada, the United Kingdom, and the United States, respectively). We present the correlations between our outcomes and the constituent measures of cognitive sophistication in the Supplemental Materials, Table S5. In a post hoc comparison of effect sizes using an *r*-to-*z* transformation, cognitive sophistication more strongly predicted misperceptions than did ideology for the U.S., *z* = 3.44, *p* < .001,^2^ and U.K. samples, *z* = 5.50, *p* < .001 (this was only marginally significant in the Canadian sample, *z* = 1.63, *p* = .052). Interestingly, cognitive sophistication was *not* a strong or consistent predictor of COVID-19 risk perceptions or behavior change intentions (Table 1).

### Identity-Protective Cognition

Next, we examined whether cognitive sophistication leads to more accurate beliefs overall, or whether it is primarily used to support motivated reasoning (thereby increasing political polarization). To test this, we interacted ideology and cognitive sophistication separately for our four dependent variables in the three samples (see Table 3). Although political ideology was a predictor of COVID-19 beliefs and misperceptions in Canada and the United States (as described above), we found no evidence whatsoever for an interaction between ideology and cognitive sophistication for any measure in any country (all β’s < .06). The result is clear: Cognitive sophistication was associated with decreased misperceptions about COVID-19 for liberals and conservatives alike (and to the same degree) in all three countries—and was unrelated to risk perceptions and prevention behavior intentions regardless of ideology.^3^

**Table 3.** Multiple Regression Analyses (*B* and 95% Confident Intervals) Interacting Political Conservatism and Cognitive Sophistication in the Prediction of our Key Dependent Variables.

Country	Misperceptions	COVID risk	Behavior changes	Leadership
Canada				
Conservatism	0.23***	−0.26***	−0.18***	−0.35***
	[0.16, 0.30]	[−0.33, −0.18]	[−0.25, −0.10]	[−0.42, −0.28]
Cognitive sophistication	−0.31***	−0.03	−0.05	−0.03
	[−0.38, −0.24]	[−0.10, 0.05]	[−0.13, 0.03]	[−0.10, 0.05]
Conservatism: CogSoph	< 0.01	−0.01	< 0.01	0.01
	[−0.07, 0.08]	[−0.08, 0.07]	[−0.08, 0.07]	[−0.06, 0.08]
*N*	644	644	644	644
*R*^2^	.17	.07	.03	.12
United Kingdom				
Conservatism	0.06	−0.04	0.06	0.44***
	[−0.01, 0.14]	[−0.12, 0.04]	[−0.02, 0.14]	[0.37, 0.51]
Cognitive sophistication	−0.39***	−0.11**	−0.09*	0.02
	[−0.47, −0.32]	[−0.19, −0.03]	[−0.17, −0.01]	[−0.05, 0.10]
Conservatism: CogSoph	−0.04	< 0.01	−0.07	0.02
	[−0.12, 0.04]	[−0.08, 0.09]	[−0.15, 0.01]	[−0.06, 0.09]
*N*	642	642	640	642
*R*^2^	.17	.01	.02	.19
United States				
Conservatism	0.24***	−0.36***	−0.17***	0.68***
	[0.17, 0.30]	[−0.43, −0.29]	[−0.24, −0.09]	[0.62, 0.73]
Cognitive sophistication	−0.42***	−0.02	−0.07	−0.02
	[−0.48, −0.35]	[−0.09, 0.05]	[−0.15, 0.00]	[−0.08, 0.03]
Conservatism: CogSoph	0.02	−0.06	−0.05	0.02
	[−0.05, 0.08]	[−0.13, 0.01]	[−0.12, 0.03]	[−0.04, 0.08]
*N*	689	689	687	689
*R*^2^	.27	.13	.03	.46

*Note*. Conservatism and cognitive sophistication were standardized (*z*-scored) within the country prior to analysis. COVID = coronavirus disease.

*** *p <* .001. **p < .01. *p < .05.

## Study 2

Study 1 found that, even in the relatively early days of the COVID-19 pandemic in the global west, political polarization was greater in the United States than in the United Kingdom and (less robustly) Canada. Despite this polarization, however, we found no evidence that cognitive sophistication was associated with stronger political polarization—contrary to the identity-protective cognition account. Indeed, cognitive sophistication was a better predictor of misperceptions than was political ideology in all three countries. There are, however, two major criticisms of these data. First, since the study was completed at the end of March, it can be argued that sufficient time had not yet passed for political polarization to take a strong hold and impact people’s thinking. Second, our measure of political ideology did not directly assess how much people *identify* with partisan groups, and thus could be considered a relatively weak proxy for political motivation. Therefore, we ran an updated version of the study, also preregistered, using the same *Prolific* quota-sampling in the United States (*N* = 697) and the United Kingdom (*N* = 641) on December 9–15, 2020.^4^ Our data, materials, and preregistration are available on the OSF. All non-preregistered analyses are labeled as post hoc.

## Method

### Participants

In total, 771 (United Kingdom), and 783 (United States) entered the survey. However, some participants did not complete the survey (*N*’s = 19, 21) and some did not indicate residing in the target country (*N*’s = 1, 1). As in Study 1, we also included three attention check questions. Following our preregistration, we removed participants who failed two or more of these (*N*’s = 108, 61). This left us with final sample sizes of 641 (United Kingdom) and 697 (United States). Full demographic breakdowns can be found in Table S1 of the Supplemental Materials.

### Materials and Procedure

Full materials and a copy of the Qualtrics survey file can be found on the OSF and descriptive statistics are available in the Supplemental Materials, Table S6. Measures were identical to Study 1, unless otherwise specified.

#### COVID-19 mitigation behaviors

Given that the survey was run 10 months into the U.S./U.K. pandemic, the relevant behavior intentions were less about behavior change intentions and more about maintenance of mitigation behaviors. In any case, these questions were administered in the same way as in Study 1 except that we updated them based on newer recommendations and asked about their intentions to engage in the behaviors. Specifically, we asked about mask wearing, limiting visits with friends and family, hand washing, avoiding public spaces that are indoors, staying home if they feel sick, and getting tested for COVID-19 if they feel sick. We also asked 10 questions about social distancing and avoiding specific public places. At the very beginning of the study, participants were asked to select “67” on a sliding scale to ensure that their browser/device allowed them to use sliders. We exclude data for the intention measure for one participant from the United Kingdom and six from the United States because they failed this check question (one who put 66 was retained). The full behavior change intention measure was reliable across both countries: Cronbach’s alpha = .90 (United Kingdom) and .92 (United States).

#### COVID-19 vaccination intentions

We added the following question: “If the federally approved COVID-19 vaccination was available to you for free, would you get vaccinated?,” with the following response options: Definitely not get it, Probably not get it, Unsure, Probably get it, Definitely get it. We also asked several exploratory questions about both COVID-19 and seasonal flu vaccines, as well as questions about past history with COVID-19 (e.g., whether they’ve contracted it). We will focus on the COVID-19 vaccination intention question for the present investigation.

#### COVID-19 risk perceptions

We decreased our number of risk items from 8 to 4 (all worded such that a higher score indicates lower risk—we reverse scored the measure for ease of interpretation). The scale had acceptable reliability in both countries: Cronbach’s alpha = .82 (United Kingdom) and .89 (United States).

#### COVID-19 misperceptions

For misperceptions, we cut the list to 12 items that were particularly salient at the time the study was run (see Table S7). We will again focus on the overall misperception measure (it was reliable in both countries: Cronbach’s α = .82 [United Kingdom] and .91 [United States]). We also added two broad misperception-like questions about whether COVID-19 is a hoax and if it has been overblown by the media. Our preregistration stated that if these correlated>.80 with the overall misperception measure, they would be added to the scale. However, they were not (*r*’s < .71).

#### Cognitive sophistication

We used the same cognitive sophistication measures as in Study 1, except the science knowledge test was shortened to eight items. Each scale had roughly acceptable reliability in both countries. CRT: Cronbach’s alpha = .69 (United Kingdom) and .74 (United States); Numeracy: Cronbach’s alpha = .66 (United Kingdom) and .71 (United States); Bullshit Receptivity: Cronbach’s alpha = .87 (United Kingdom) and .89 (United States); Science knowledge: Cronbach’s alpha = .67 (United Kingdom) and .68 (United States). As in Study 1, we report the overall results with the omnibus measure of cognitive sophistication. The reliability of the four measures was similar to the reliabilities for the individual subscales in each country (weighing each subscale equally): Cronbach’s alpha = .69 (United Kingdom) and .67 (United States).

#### Trust

As in Study 1, we asked the participants to indicate their degree of trust in various information sources using a 5-point scale from “none at all” to “a great deal.” Unlike in Study 1, we preregistered an analysis using the media trust questions. In particular, we first correlated political conservatism (described below) with trust in the various news outlets and then created two composite measures based on the pattern of correlations. Those outlets that conservatives trusted more were coded as “conservative media” (United States: Fox News, Breitbart, Newsmax, One American News; United Kingdom: Daily Mail, The Times, The Daily Telegraph, and The Sun) and those outlets that liberals trusted more were coded as “liberal media” (United States: CNN, MSNBC, New York Times, Washington Post, NPR, CBS News, ABC News, NBC News; United Kingdom: BBC, The Guardian, Channel 4). There were three outlets that did not correlate with ideology in the U.K. sample (Metro, Sky News, ITV News) and they were not included in our analyses. All four measures had good reliability: Cronbach’s alpha = .96 (liberal media, United States), .83 (liberal media, United Kingdom), .87 (conservative media, United States), .82 (conservative media, United Kingdom).

#### Political ideology and partisan identification

Our ideology measures were the same as in Study 1, with a few exceptions. First, we revised the ideology question so that it said “left/liberal” and “right/conservative” to clarify the nature of the question. Second, we asked participants in the United States about who they voted for in the 2020 election (and what method they used). This is an exploratory measure. Second, and more importantly, we included a partisan identification measure (Leach et al., 2008). The scale included 14 questions, such as “I feel solidarity with (selected Political Party.” Given that there are multiple political parties in the United Kingdom and that this measure was included to more directly test the identity-protective cognition account [which makes a stronger prediction in the United States than the United Kingdom], we preregistered that we would focus our partisan identification measure on American individuals who identify with the Democratic or Republican parties.^5^ Finally, we removed a constant of 8 for Democrats to reverse score the mean. We then added a constant of 7 for Republicans so that the full-scale mean would vary continuously from 1 (indicating strongest identification with the Democratic Party) to 14 (indicating strongest identification with the Republican Party). Re-coding the identification measure in this way allows for a more direct comparison between the measure and our other measures of political ideology and partisanship (which all vary continuously from, for example, strongly liberal to strongly conservative). Collapsing or “unfolding” the measure in this way has precedent in studies looking at ideological intensity (e.g., Huddy et al., 2018) and is akin to unfolding across political consistency to avoid an extraneous interaction term in the model (e.g., Pennycook & Rand, 2019b) or re-coding measures so that identification matches with the outcome (Hackel et al., 2014). The scale had high reliability across party lines: Cronbach’s alpha = .95 (Democrats) and .97 (Republicans).

## Results

### Political Ideology

**Figure 1.**
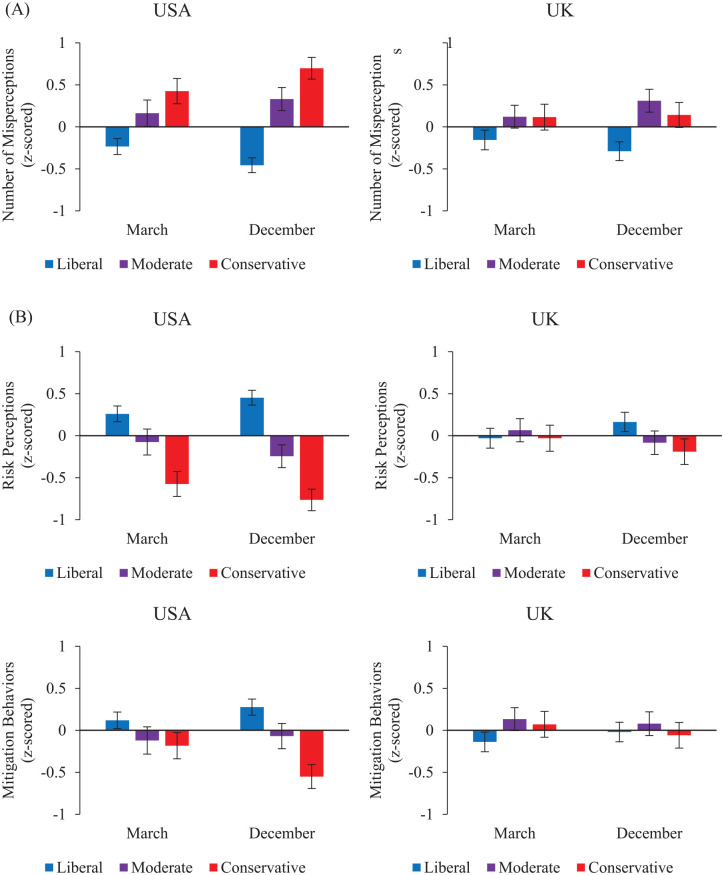
Change in relative polarization in COVID-19 misperceptions (A), risk perceptions (B), and mitigation behaviors (C) from March (Study 1) to December (Study 2) in the United States and the United Kingdom. Means were standardized (*z*-scored), such that a value of 1 indicates 1 standard deviation above or below the mean. Political polarization was much stronger in the United States relative to the United Kingdom, and this increased from March to December. Error bars are 95% confidence intervals. COVID-19 = coronavirus disease 2019.

To investigate the differential impact of political polarization, we correlated political ideology with our COVID-19 belief and behavior measures across both countries. As is evident from Table 4, political conservatism was associated with misperceptions in both countries. However, this correlation was over twice as strong in the United States than in the United Kingdom (Table 5). The same pattern was held for perceptions of COVID-19 risk, vaccination intentions, and behavioral intentions (e.g., mask wearing, social distancing). Ideology was also a stronger predictor of COVID-19 risk perceptions in the United States than in the United Kingdom once demographics (age, gender, income, education, and overall health) were controlled for (see Table S8 in Supplemental Materials—all other findings were identical with demographics included). The party identification measure had similar correlations as political ideology in the U.S. sample. As is evident in Figure 1, political polarization was much stronger in the United States relative to the United Kingdom, and this increased from March (Study 1) to December (Study 2).

**Table 4.** Zero-Order Correlations Between Primary Measures in the United Kingdom (*N* = 641) and the United States (*N* = 697).

Country	Variable	1	2	3	4	5	6	7	8
United Kingdom	1. Misperceptions	—							
	2. COVID risk	−.47***	—						
	3. Vaccination intentions	−.53***	.38***	—					
	4. Mitigation behaviors	−.31***	.48***	.31***	—				
	5. Conservatism	.21***	−.18***	−.06	−.02	—			
	6. Conservative media trust	.01	.04	.07	.03	.29***	—		
	7. Liberal media trust	−.31***	.28***	.30***	.14***	−.16***	.53***	—	
	8. Cognitive sophistication	−.43***	.08*	.22***	−003	−.13**	−.08*	.14***	—
United States	1. Misperceptions	—							
	2. COVID risk	−.72***	—						
	3. Vaccination intentions	−.55***	.47***	—					
	4. Mitigation behaviors	−.52***	.59***	.47***	—				
	5. Conservatism	.51***	−.54***	−.35***	−.36***	—			
	6. Conservative media trust	.48***	−.43***	−.16***	−.16***	.47***	—		
	7. Liberal media trust	−.42***	.39***	.42***	.37***	−.43***	−.05	—	
	8. Cognitive sophistication	−.35***	.17***	.21***	.07	−.14***	−.27***	.03	—
	9. Party identification	.42***	−.47***	−.34***	−.36***	.76***	.39***	−.58***	−.03

*Note.* Party identification is scored such that a higher score indicates a stronger identification with the Republican Party and a lower score indicates a stronger identification with the Democratic Party (and is therefore restricted to individuals who identify with either the Democratic or Republican Party). COVID = coronavirus disease.

*** *p* < .001. **p < .01. *p < .05.

**Table 5.** Multiple Regression Analyses (*B* and 95% Confident Intervals) Comparing the Correlation Between Key Dependent Variables and Ideology Across Countries (With the United States as a Baseline).

Country	Misperceptions	COVID risk	Vaccination intention	Mitigation behaviors
Conservatism	0.51***	−0.54***	−0.35***	−0.36***
	[0.44, 0.58]	[−0.61, −0.47]	[−0.42, −0.28]	[−0.43, −0.29]
Conservatism: United Kingdom	−0.21***	0.25***	0.20***	0.23***
	[−0.40, −0.20]	[0.26, 0.45]	[0.19, 0.40]	[0.23, 0.44]
*N*	1,338	1,338	1,338	1,334
*R* ^2^	.16	.17	.07	.07

*Note.* Conservatism was standardized (*z*-scored) within country prior to analysis. United Kingdom (*N* = 641), United States (*N* = 697). COVID = coronavirus disease.

As in Study 1, there was a divergence between the countries in terms of trust in the news media. In both countries, trust in liberal media outlets was an association with lower misperceptions, stronger intentions to engage in appropriate behaviors and to get vaccinated, and a stronger sense of the risk of COVID-19 (although these correlations tended to be stronger in the United States). As in Study 1, trust in “conservative” media outlets was generally not associated with our key dependent variables in the United Kingdom but was strongly associated with COVID misperceptions and skepticism in the United States Furthermore, as in Study 1, media trust was, if anything, a stronger predictor than political ideology for misperceptions in the United States. In fact, conservative media trust was twice as strong of a predictor of misperceptions than political ideology in a multiple regression analysis (that also included liberal media trust): liberal media trust: β = −.33, *p* < .001, conservative media trust: β=.38, *p* < .001, conservatism: β = .18, *p* < .001.^6^ This accords with the idea that political polarization in the United States (but not the United Kingdom) has been—at least in part—driven by the often problematic coverage of the issue in the conservative media ecosystem (Gollwitzer et al., 2020; Simonov et al., 2020).

### Cognitive Sophistication

As is evident from Table 4, overall cognitive sophistication (see Supplemental Materials for subscale analysis; Table S9) was a strong negative predictor of COVID-19 misperceptions (*r*’s = −.43, −.36 in the United Kingdom and the United States, respectively) and, unlike Study 1, also correlated (albeit weakly) with stronger risk perceptions (*r*’s = .08, 17. in the United Kingdom and the United States, respectively). In the U.K. sample, cognitive sophistication more strongly predicted misperceptions than ideology, *z* = 4.53, *p* < .001, and the same was true for vaccination intentions, *z* = 3.15, *p* = .001 (these were post hoc tests^7^). However, unlike Study 1, this was *not* the case in the U.S. sample—in fact, conservatism was a *stronger* predictor than cognitive sophistication for both misperceptions, *z* = 3.51, *p* < .001, and vaccination intentions, *z* = 2.95, *p* = .002 (these were also post hoc tests). This is apparently a consequence of the increased political polarization in the United States in December relative to March (see Figure 1).

### Identity-Protective Cognition

The foregoing suggests that COVID-19, as of December 2020, represents an even *stronger* test case for the identity-protective cognition account than it was in Study 1. For this, we interacted ideology and cognitive sophistication separately for our four key dependent variables—focusing on the more polarized context of the United States (see Table 6). As noted earlier, we administered an additional measure of party identification as a stronger test for the predicted interaction between cognitive sophistication and partisanship (in predicting COVID-19 beliefs and behaviors; see Table 6).

**Table 6.** Multiple Regression Analyses (*B* and 95% Confident Intervals) Interacting Political Conservatism and Cognitive Sophistication in the Prediction of our Key Dependent Variables (U.S. Data Only).

Variable	Misperceptions	COVID risk	Vaccination intentions	Mitigation behaviors
Conservatism	0.47***	−0.53***	−0.33***	–0.36***
	[0.40, 0.53]	[−0.59, −0.47]	[−0.40, −0.26]	[−0.43, −0.29]
Cognitive sophistication	−0.29***	0.09**	0.17***	0.02
	[−0.35, −0.23]	[0.03, 0.16]	[0.10, 0.24]	[−0.06, 0.09]
Conservatism: CogSoph	−0.01	−0.06*	−0.05	−0.10**
	[−0.07, 0.05]	[−0.13, 0.00]	[−0.12, 0.02]	[−0.18, −0.03]
*N*	697	697	697	693
*R* ^2^	.34	.30	.15	.14
Party identification	0.43***	−0.50***	−0.32***	−0.36***
	[0.36, 0.50]	[−0.57, −0.43]	[−0.40, −0.25]	[−0.44, −0.29]
Cognitive sophistication	−0.33***	0.15***	0.18***	0.04
	[−0.40, −0.27]	[0.09, 0.22]	[0.11, 0.25]	[−0.04, 0.11]
Party ID: CogSoph	0.11**	−0.17***	−0.02	−0.13***
	[0.04, 0.18]	[−0.24, −0.10]	[−0.10, 0.05]	[−0.20, −0.06]
*N*	591	591	591	587
*R* ^2^	.34	.27	.15	.15

*Note.* Measures were standardized (*z*-scored) prior to analysis.

*** *p <* .001. **p < .01. *p < .05.

First, there were no interactions between ideology/identity and cognitive sophistication for vaccination intentions, indicating that cognitive sophistication is associated with a stronger likelihood of getting vaccinated for liberals/Democrats and conservatives/Republicans alike. However, there were some significant interactions with the other measures (and, most notably, with political identity for misperceptions). To clarify the underlying interactions, Table 7 reports the correlation between cognitive sophistication and the four COVID-19 measures separately for strong Democrats and strong Republicans (as determined by having a mean score above “agreement” on the identification scale for the respective groups—this was a post hoc analysis). This analysis shows, consistent with Study 1, that cognitive sophistication is associated with decreased misperceptions about COVID-19 for both groups; however, this correlation is notably weaker for strong Republicans than it is for strong Democrats.^8^ Furthermore, COVID-19 risk perceptions and behavior intentions were nominally *negatively* correlated with cognitive sophistication among strong Republicans (although these correlations were not significant). Thus, although cognitive sophistication is not significantly associated with beliefs and behaviors about COVID-19, political partisanship does seem to be counteracting and undermining the influence of strong reasoning skills.

**Table 7.** Zero-Order Correlations Between Cognitive Sophistication and Primary Measures Among Relatively Strong Democrats (*N* = 208) and Strong Republicans (*N* = 127), Based on Party Identification.

Variable	Misperceptions	COVID risk	Vaccination intentions	Mitigation behaviors
Strong democrats	−.43***	.32***	.18*	.24**
Strong republicans	−.21*	−.07	.13	−.17

*Note.* COVID = coronavirus disease.

The foregoing is broadly consistent with the identity-protective cognition account. However, given the strength of the correlations with media trust relative to political ideology and partisan identification in the United States, we also performed a post hoc test including media trust as a control (see Table 8). This analysis revealed that partisan identification did *not* interact with cognitive sophistication once liberal and conservative media trusts (and their interactions with cognitive sophistication) were included in the regression for misperceptions. There was similarly no interaction between partisan identification and cognitive sophistication for vaccination intentions or mitigation behaviors (although it did remain for risk perceptions). In fact, the most consistent predictor across our measures was trust in liberal media outlets.

**Table 8.** Multiple Regression Analyses (*B* and 95% Confident Intervals) Interacting Political Conservatism and Media Trust With Cognitive Sophistication in the Prediction of our Key Dependent Variables (U.S. Data Only).

Variable	Misperceptions	COVID risk	Vaccination intentions	Mitigation behaviors
Party identification	0.11**	−0.27***	−0.09	−0.21***
	[0.03, 0.19]	[−0.36, −0.18]	[−0.19, 0.01]	[−0.31, −0.11]
Cognitive sophistication	−0.24***	0.09**	0.14***	0.01
	[−0.30, −0.18]	[0.03, 0.16]	[0.07, 0.22]	[−0.07, 0.09]
Party ID: CogSoph	0.08	−0.12**	−0.01	−0.07
	[−0.002, 0.16]	[−0.21, −0.03]	[−0.11, 0.09]	[−0.17, 0.03]
Liberal media trust	−0.31*** [−0.38, −0.24]	0.23*** [0.15, 0.30]	0.30*** [0.21, 0.38]	0.19*** [0.10, 0.27]
Liberal media trust: CogSoph	0.04 [−0.03, 0.11]	0.02 [−0.06, 0.09]	−0.08 [−0.16, 0.01]	0.03 [−0.05, 0.12]
Conservative media trust	0.34*** [0.27, 0.40]	−0.26*** [−0.33, −0.18]	−0.10* [−0.19, −0.02]	−0.07 [−0.16, −0.01]
Conservative media trust: CogSoph	−0.12*** [−0.18, −0.06]	0.13*** [0.06, 0.19]	−0.05 [−0.13, 0.03]	−0.03 [−0.11, 0.05]
*N*	590	590	590	586
*R* ^2^	.49	.39	.22	.18

*Note*. Measures were standardized (*z*-scored) prior to analysis.

*** *p <* .001. **p < .01. *p < .05.

## Discussion

Anecdotal evidence indicates that partisanship has played a more significant role in the early stages of the public discourse surrounding the COVID-19 pandemic in the United States than in the United Kingdom. Our results support this conclusion: Political ideology was a stronger predictor of beliefs and attitudes relating to COVID-19 (including risk perceptions, behavior change intentions, misperceptions, and support for national leadership) in the United States than in the United Kingdom. Interestingly, Canada and the United States were more similar although there was nonetheless some evidence that polarization was greater in the United States Furthermore, polarization seems to have increased markedly in the interim between March and December 2020, particularly in the United States.

### Consequences of Analytic Thinking

Despite this political polarization, cognitive sophistication—that is, the quality of one’s reasoning—was consistently associated with lower misperceptions. In the United Kingdom and in the early survey in the United States, cognitive sophistication was a stronger predictor of resistance to misperceptions than was political ideology. Although this reversed by December in the United States, we found no evidence in any country that cognitive sophistication was associated with *stronger* misperceptions—thus, at least in terms of avoiding falsehoods about COVID-19, improving scientific literacy and reasoning skills seems an important pathway for inoculation against misinformation even in the face of political polarization. Still, the contrast between the March and December studies illustrates how increasing polarization in public discourse can undermine the influence of reasoning skills—in particular, the correlation between cognitive sophistication and our various outcome measures was weaker among people who identified with the Republican Party following around 10 months of political polarization.

This research resonates with an important claim of science deficit models: That teaching people the basics of science will lead to more acceptance and positive attitude change. There is some evidence demonstrating the effectiveness of this approach with genetically modified foods (McPhetres et al., 2019b) and climate change (Ranney & Clark, 2016). However, it is also clear that even if this is effective in the aggregate, it is likely to be undermined if political polarization is sufficiently strong.

We also observed an interesting pattern that both supports the conclusions of this research and suggests limits to it: Greater levels of cognitive sophistication were strongly associated with reduced levels of misconceptions and stronger vaccination intentions, but not with behavior change intentions or mitigation behaviors. This accords with recent work showing that conspiratorial ideation and belief in pseudoscience did not relate to compliance to official COVID-19 recommendations (Díaz & Cova, 2020). This seems to suggest that being reflective, numerate, skeptical, and having basic science knowledge (or some combination of these things) is important for the ability to identify false information about the virus, but it may not be enough to determine what behaviors are most effective or to motivate one to change their behaviors (apart from those that are strongly linked to misperceptions, such as vaccination intentions).

The foregoing highlights some interesting questions with respect to effective science communication. Given the complexity and level of uncertainty regarding the risks, dangers, and future outcomes of the COVID-19 pandemic, science communicators may wish to focus on communicating consistent behaviors rather than teaching people how the virus works or trying to provide information on risk levels, transmission, or other complex factors. Particularly given the current uncertainty, clear and consistent messages regarding what people should and shouldn’t do seems paramount.

### Evaluating the Evidence for Identity-Protective Cognition

Our data indicate parallels between COVID-19 and global warming. Specifically, global warming is one of the few scientific issues where beliefs are more consistently predicted by political ideology (in the United States) than cognitive sophistication (McPhetres et al., 2019a). Although this was not apparently the case early on in the pandemic (Study 1)—or even later on in the United Kingdom—politicization of COVID apparently increased to such an extent that (in the United States) ideology and partisan identification became stronger predictors of misperceptions than reasoning skills. Furthermore, we found an interaction between cognitive sophistication and partisan identification among U.S. participants in Study 2, indicating that reasoning skills were more weakly related to having accurate COVID-19 beliefs among Republicans. This is a common finding for climate change as well (Kahan et al., 2012, 2017; McPhetres, Bago, & Pennycook, 2019; Pennycook et al., 2020).

Given that identity-protective cognition is a favored explanation for polarization around climate change (Kahan et al., 2012, 2017), do our results indicate that ideology plays a causal role in the formation of false beliefs about COVID-19? There is some reason to be skeptical of this claim. First, trust in news media was a more consistent predictor of misperceptions than political ideology or partisan identification in the United States. In fact, in Study 2 when polarization was the greatest, both conservative and liberal media trust were three times as strong of predictors of misperceptions than partisan identification. This is consistent with recent research showing that engagement with conservative media in the United States (and Fox News, in particular) is associated with reduced physical distancing (Gollwitzer et al., 2020; Simonov et al., 2020). If partisans engage with different media sources, it is possible that polarization is not a result of identity-protective cognition per se, but rather differential exposure to different messaging about COVID-19 (for further discussion of the confounded nature of group difference comparisons in the context of partisan bias, see Druckman and McGrath (2019) and Tappin et al. (2020c). However, it is important to note that our media trust analyses were exploratory and it is difficult to disentangle partisanship from media trust in a highly fractured political information environment such as the United States. Future work is needed to determine which factors are playing the strongest causal role.

Nonetheless, in further support of this perspective, recent research has shown that cases, where cognitive sophistication is associated with increased polarization, may be attributable to differences in prior factual beliefs (Tappin et al., 2020a, 2020b). For example, a recent study found that experimentally manipulating reasoning led people to increase the coherence between their prior beliefs about climate change and their evaluation of arguments for or against anthropogenic global warming (Bago et al., 2020b). In short, cognitive sophistication may interact with polarization simply because people who tend to be more willing to engage in analytic thinking are more likely to have strong prior beliefs about political topics. Given that it is not irrational to consider priors when updating beliefs (e.g., Gerber & Green, 1999), partisan “bias” could emerge from differences in information environments and even absent any causal influence of political identities on cognitive processing. Of course, the question of how our identities influence selection into information environments is a critical one.

## Conclusion

The COVID-19 pandemic reveals a great deal about the strengths and weaknesses of human psychology. Social scientists have a responsibility to learn as much as possible about people’s beliefs, attitudes, and behaviors relating to the global pandemic so that we can be more prepared the next time that humanity has to face similar struggles. The present work indicates not only that political polarization can occur quite rapidly and even in the face of a collective crisis (as in the United States) but also that it is not inevitable (as in the United Kingdom). Furthermore, at least in terms of misperceptions, our findings further support past research on the importance of nurturing competency in cognitive processing as a pre-inoculation against polarized messaging from political elites and vested interests. However, our results also highlight how particularly strong political polarization can blunt the positive effects of analytic thinking—thus, improving the quality of people’s thinking without addressing the underlying political polarization may have limited effectiveness in some cases. Future research should continue to track these developments and further investigate pragmatic long-term interventions that can increase people’s basic reasoning competencies in the face of political polarization.

## Supplemental Material

sj-docx-1-psp-10.1177_01461672211023652 – Supplemental material for Beliefs About COVID-19 in Canada, the United Kingdom, and the United States: A Novel Test of Political Polarization and Motivated Reasoning Supplemental material, sj-docx-1-psp-10.1177_01461672211023652 for Beliefs About COVID-19 in Canada, the United Kingdom, and the United States: A Novel Test of Political Polarization and Motivated Reasoning by Gordon Pennycook, Jonathon McPhetres, Bence Bago and David G. Rand in Personality and Social Psychology Bulletin

## Notes

1 Collinearity does not appear to be a problem here, variance inflation factor (VIF) for conservatism = 1.78. The parallel analysis using political partisanship produces the same result: liberal media trust: β = −.13, *p* = .002, conservative media trust: β = .35, *p* < .001, Republican partisanship: β = .05, *p* = .288.

2 The parallel analysis comparing cognitive sophistication and political partisanship in the United States produced the same result (the former being a stronger predictor of misperceptions than the latter): *z* = 3.29, *p* < .001.

3 We repeated this analysis using our continuous political partisanship measure in the U.S. sample, and it produced the same null interactions (all β’s < .04, *p*’s > .340). Post hoc tests also revealed that neither liberal (β = −.03, *p* = .318) nor conservative (β = −.01, *p* = .712) media trust significantly interacted with cognitive sophistication when predicting misperceptions in the United States.

4 Both samples were launched on December 9; however, a glitch in the *Prolific* website paused the U.K. sample after ~160 individuals completed the survey and the problem was not fixed until December 14, at which time we simply re-ran the entire batch from the United Kingdom starting on the 15th (this was necessary to maintain the quota-sampling).

5 We asked individuals who initially identified as “Independent” to determine if they “Lean Democrat,” “Lean Republican” or are “True Independents”—those who leaned in one direction were included in the larger set of Democrats/Republicans for the analysis.

6 The parallel analysis using party identification produces a similar result: liberal media trust: β = −.33, *p* < .001, conservative media trust: β = .49, *p <* .001, Republican Party identification: β = .05, *p* = .264.

7 We forgot to preregister it. In any case, this was a replication of an analysis reported in Study 1.

8 A parallel analysis separating participants based on a single-item affiliation with political party question produced a similar result. Correlation between cognitive sophistication and misperceptions: Democrats, *r*(350) = −.40, *p <* .001; Republicans, *r*(136) = −.17, *p* = .046; Independents, *r*(181) = −.45, *p <* .001.
