# Supplementary material for: Beliefs About COVID-19 in Canada, the United Kingdom, and the United States: A Novel Test of Political Polarization and Motivated Reasoning
Source: Pers Soc Psychol Bull. 2021 Jun 28;48(5):750–65. doi: 10.1177/01461672211023652 (PMC9066691; doi:10.1177/01461672211023652)
Supplement: sj-docx-1-psp-10.1177_01461672211023652 – Supplemental material for Beliefs About COVID-19 in Canada, the United Kingdom, and the United States: A Novel Test of Political Polarization and Motivated Reasoning [file sj-docx-1-psp-10.1177_01461672211023652.docx]

**Methodology File**

for

**Beliefs about COVID-19 in Canada, the U.K., and the U.S.A.:**

**A novel test of political polarization and motivated reasoning**

**Explain how sample size was determined, including whether and how looking at the results influenced the collection of additional data. If sample size was determined ahead of time using a power analysis, please report the basis for the expected effect size and all information required to reproduce the power analysis.**

We did not complete a power analysis ahead of time. Rather, we determined the maximum amount we were willing to spend on the study and chose 750 per sample. This was preregistered for both studies.

**Report (1) the total number of excluded observations, (2) the reasons for making these exclusions, (3) how they were distributed across conditions.**

Participants who failed attention checks were excluded. Study 1: “We recruited 750 participants each in Canada, the U.K., and the U.S. via Prolific on March 24th, 2020. The U.K. and U.S. samples were recruited with quota-matching to approximate the national populations (via census data) across age, sex, and ethnicity. Prolific does not offer quota-matching for Canadian samples. In total, 753 (Canada), 765 (U.K.), and 759 (U.S.) entered the survey. However, some participants did not complete the survey (N’s = 5, 11, 17) and some did not indicate residing in the target country (N’s = 2, 1, 1). We also included 3 attention check questions (see [OSF](https://osf.io/3a497/?view_only=73414de50492413f9c081c4a0e0d7f42) for full materials). Following our preregistration, we removed participants who failed 2 or more of these (N’s = 104, 111, 52). This left us with final sample sizes of 644 (Canada), 642 (U.K.), and 689 (U.S.). Study 2: We recruited 750 participants in the U.K. and the U.S. via Prolific on December 9^th^ and 15^th^, 2020. The samples were recruited with quota-matching to approximate the national populations (via census data) across age, sex, and ethnicity. In total, 771 (U.K.), and 783 (U.S.) entered the survey. However, some participants did not complete the survey (N’s = 19, 21) and some did not indicate residing in the target country (N’s = 1, 1). As in Study 1, we also included 3 attention check questions. Following our preregistration, we removed participants who failed 2 or more of these (N’s = 108, 61). This left us with final sample sizes of 641 (U.K.) and 697 (U.S.).

**Disclose the existence of all variables and conditions that were part of the study. These can be summarized, or put in a footnote or supplementary material in the case of large numbers of variables, but there should be enough information for a reader to judge whether the variables and/or conditions are potentially theoretically relevant or not.**

All measures are disclosed in the methods sections of the manuscript. Verbatim copies of the materials can be found on [OSF](https://osf.io/3a497/?view_only=73414de50492413f9c081c4a0e0d7f42).
